# Cryo-EM structure of SKP1-SKP2-CKS1 in complex with CDK2-cyclin A-p27KIP1

**DOI:** 10.1038/s41598-023-37609-9

**Published:** 2023-07-03

**Authors:** Rhianna J. Rowland, Richard Heath, Daniel Maskell, Rebecca F. Thompson, Neil A. Ranson, James N. Blaza, Jane A. Endicott, Martin E. M. Noble, Marco Salamina

**Affiliations:** 1 Translational and Clinical Research Institute, Newcastle University Centre for Cancer, Newcastle University, Paul O’Gorman Building, Framlington Place, Newcastle Upon Tyne, NE2 4HH UK; 2 Astbury Centre for Structural Molecular Biology, School of Molecular and Cellular Biology, University of Leeds, Leeds, LS2 9JT UK; 3 Department of Chemistry, York Structural Biology Laboratory and York Biomedical Research Institute, University of York, Heslington, YO10 5DD York UK; 4 Present Address: Life Sciences Electron Microscopy, Thermo Fisher Scientific, Leeds, UK; 5 Present Address: Evotec (UK) Ltd., Milton, Abingdon, OX14 4RZ UK

**Keywords:** Biochemistry, Structural biology

## Abstract

p27KIP1 (cyclin-dependent kinase inhibitor 1B, p27) is a member of the CIP/KIP family of CDK (cyclin dependent kinase) regulators that inhibit cell cycle CDKs. p27 phosphorylation by CDK1/2, signals its recruitment to the SCF^SKP2^ (S-phase kinase associated protein 1 (SKP1)-cullin-SKP2) E3 ubiquitin ligase complex for proteasomal degradation. The nature of p27 binding to SKP2 and CKS1 was revealed by the SKP1-SKP2-CKS1-p27 phosphopeptide crystal structure. Subsequently, a model for the hexameric CDK2-cyclin A-CKS1-p27-SKP1-SKP2 complex was proposed by overlaying an independently determined CDK2-cyclin A-p27 structure. Here we describe the experimentally determined structure of the isolated CDK2-cyclin A-CKS1-p27-SKP1-SKP2 complex at 3.4 Å global resolution using cryogenic electron microscopy. This structure supports previous analysis in which p27 was found to be structurally dynamic, transitioning from disordered to nascent secondary structure on target binding. We employed 3D variability analysis to further explore the conformational space of the hexameric complex and uncovered a previously unidentified hinge motion centred on CKS1. This flexibility gives rise to open and closed conformations of the hexameric complex that we propose may contribute to p27 regulation by facilitating recognition with SCF^SKP2^. This 3D variability analysis further informed particle subtraction and local refinement approaches to enhance the local resolution of the complex.

## Introduction

The cyclin-dependent protein kinases (CDKs), CDK1, CDK2 and CDK4/6 coordinate events of the eukaryotic cell cycle to ensure genome and cell integrity is maintained through generations^1–3^. Activation of CDK4/6-cyclin D complexes is a response to mitogenic signals early in G1, and cell cycle progression is sustained through late G1 and S-phase by CDK2 partnered first by cyclin E and then cyclin A. CDK1-cyclin B is also reported to be active during S-phase^4^ and the activity of CDK1 bound to cyclins A and B guides the cell through G2 into mitosis to ensure its timely completion. The CDK-cyclin module is regulated by reversible phosphorylation and association with a range of protein inhibitors and activators that modulate activity, substrate preferences and intracellular location (reviewed in^5,6^). Their timely destruction by the ubiquitin proteasome system is essential to CDK control of the cell cycle, and their selection is dependent on a number of substrate-specific E3 ligases^7^.p27KIP1 (cyclin-dependent kinase inhibitor 1B, p27) is a member of the CIP/KIP family of CDK regulators that inhibit cell cycle CDKs^8^. p27 is also a CDK4/6-selective assembly factor that promotes association with D-cyclins^8–15^. A variety of signals increase p27 levels to cause growth arrest, including cytokines, such as transforming growth factor β (TGFβ)^16,17^ and interferon-γ (IFN-γ)^18^; and inducers of differentiation such as vitamin D3 and retinoic acid^19^. In contrast, low p27 levels are commonly identified in cancer and associated with poor prognosis^20,21^. p27 is degraded via the ubiquitin proteasome system^22,23^, where phosphorylation at Thr187 by CDK1/2 signals its recruitment to the S-phase kinase associated protein 1 (SKP1)-cullin-SKP2 E3 ubiquitin ligase complex SCF^SKP2^^24–31^. Though best characterised as E3 subunits, SKP1 and SKP2 were initially identified as part of a pentameric CDK2-cyclin A-CKS1-SKP1-SKP2 complex^32–34^. Notably, p27 bound to a CDK-cyclin module is the SCF^SKP2^ substrate and unusually the binding site for phosphorylated p27 is contributed by both SKP2 and the accessory protein, CKS1^26,35–38^. The nature of this interaction was revealed through a SKP1-SKP2-CKS1-p27 phosphopeptide crystal structure^38^. Given its cellular importance in regulating p27, a predicted model for the CDK2-cyclin A-CKS1-p27-SKP1-SKP2 complex was proposed by overlaying independent structures of CDK2-cyclin A-p27^39^ and SKP1-SKP2-CKS1-p27 peptide^40^. However, definitive experimental evidence for this architecture has not yet been reported.

Here we report an experimentally determined structure of the isolated CDK2-cyclin A-CKS1-p27-SKP1-SKP2 complex using cryogenic electron microscopy (cryo-EM). At a global resolution of 3.4 Å (Fourier Shell Correlation (FSC) 0.143 cut-off), we provide structural analyses of the overall quaternary architecture and interactions that underpin this complex. The structure is in broad agreement with the octameric CUL1-RBX1-SKP1-SKP2-CKSHS1-cyclin A-CDK2-p27 complex (PDB **7B5R**) which was recently determined by cryo-EM as a focussed refined sub-complex of the full SCF-RBR E3-E3 super-assembly. We further enhanced our EM map of the hexameric complex through particle subtraction and local refinement to reveal additional structural features. 3D variability analysis also uncovered a significant hinge motion centred on CKS1, which gives rise to open and closed conformations of the complex. We speculate this motion may contribute to ubiquitin-mediated degradation of p27 by facilitating substrate recognition with the SKP2 recognition motif of SCF^SKP2^.

## Results and discussion

### The structure of the CDK2-cyclin A-CKS1-SKP1-SKP2-p27 complex

**Figure 1:**
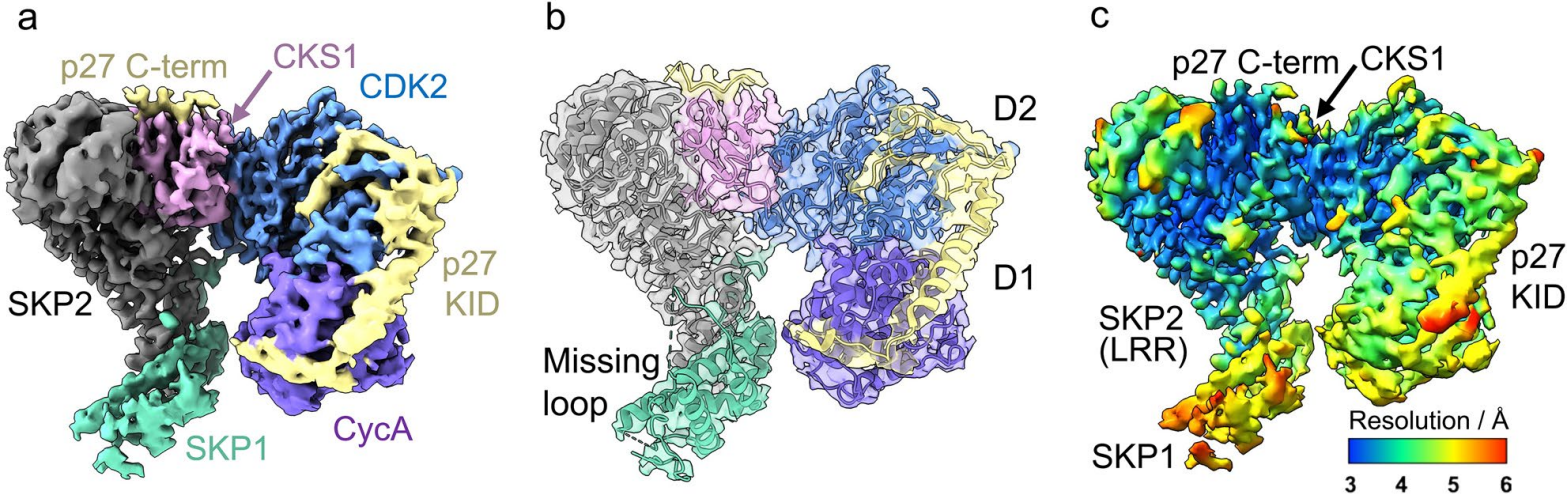
3.4 Å Cryo-EM map of the hexameric SKP1-SKP2-CKS1-CDK2-cyclin A-p27 complex. (**a**) 3.4 Å cryo-EM map (PDB **8BYA**, EMD-**16325**) into which the full hexameric complex, comprised of SKP1 (green), SKP2 (grey), CKS1 (pink), CDK2 (blue), cyclin A (purple) and p27 (yellow), was fitted and refined using starting models PDB **2AST** and **1JSU**. (**b**) The refined model shows domain 1 (D1) and the helical linker domain of the N-term kinase inhibitory domain (KID) of p27 wrapping around cyclin A and engaging with the RxL binding motif whilst domain 2 (D2) extends over CDK2 towards the activation segment. The C-terminal domain of p27 lies over CKS1. (**c**) Local resolution analysis of the hexameric complex revealing highest resolution within SKP2(LRR)-CKS1 core (~ 3–3.5 Å) and lowest resolution (~ 5–6 Å) in the outer region of the p27 KID and SKP1.

Following screening by negative stain transmission electron microscopy, the hexameric complex was imaged by cryo-EM and refined to a global resolution of 3.4 Å (FSC 0.143) (Fig. 1, Supplementary Figs. S1–S4, Supplementary Table S1, PDB ID **8BYA**, EMD-**16325**). The data processing workflow is described in Supplementary Figs. S2 and S3. In summary, an initial non-biased particle set was picked using blob picker and used to generate 2D classes that were lowpass filtered to 20 Å (to reduce template bias) as templates for picking. An ab-initio 3D reconstruction was generated and used to create new 2D templates for further picking. Template picked particles were subjected to heterogeneous refinement using three 3D classes, with refinement of the best class (136,325 particles) yielding the final 3D reconstruction at 3.4 Å. An initial model for the hexameric complex was generated in UCSF ChimeraX^41,42^ by rigid body docking CDK2-cyclinA-p27 (PDB **1JSU**) and SKP1-SKP2-CKS1-p27 (PDB **2AST**) to give an initial model-to-map fit CCmask = 0.49. The fitting of the model to the EM map was improved (CCmask = 0.73) using real-space refinement^43^ in Phenix and manual model building in COOT^44^ (RMSD = 2.3 of all residues between initial and refined model). This refinement left SKP2 and CKS1 relatively unchanged compared to PDB **2AST**, whilst moderate changes to the SKP1 domain and conformation of p27 KID bound to CDK2-Cyclin A were required (Supplementary Fig. S4c, described in more detail below).

The EM map covers the full-length of CDK2 and CKS1, and the expressed residues of cyclin A (174–432) that compose the tandem cyclin box folds. SKP1, SKP2 and p27 were expressed as full-length proteins but their structures could not be fully modelled. SKP1 has two disordered loop regions at residues 31–41 (loop 1) and 64–79 (loop 2), whilst the SKP2 structure starts at Val95 and includes the F-box (residues 109–151), leucine rich repeats (LRR, residues 151–402) and C-terminal tail (residues 402–419).

Previous studies have indicated that the N-terminus of SKP2 binds to cyclin A^45^. However, this interaction is known to be mutually exclusive of p27 binding and a model in which p27 and SKP2 both engage with the cyclin A RxL site, precluding simultaneous binding in this region, has been proposed^46,47^. Consequently, we do not observe this interaction in the hexameric complex. The lack of density for the N-terminal SKP2 residues 1–94 suggests it may be disordered in this complex. In the case of p27, the kinase inhibitory domain (KID, residues 25–93) and C-terminal sequence surrounding the phosphorylated Thr187 residue (residues 181–190) were modelled but the linking residues (94–180) were not visible (Fig. 1b). Equivalent disordered regions were noted in the octameric complex (PDB **7B5R**^48^).

**Figure 2:**
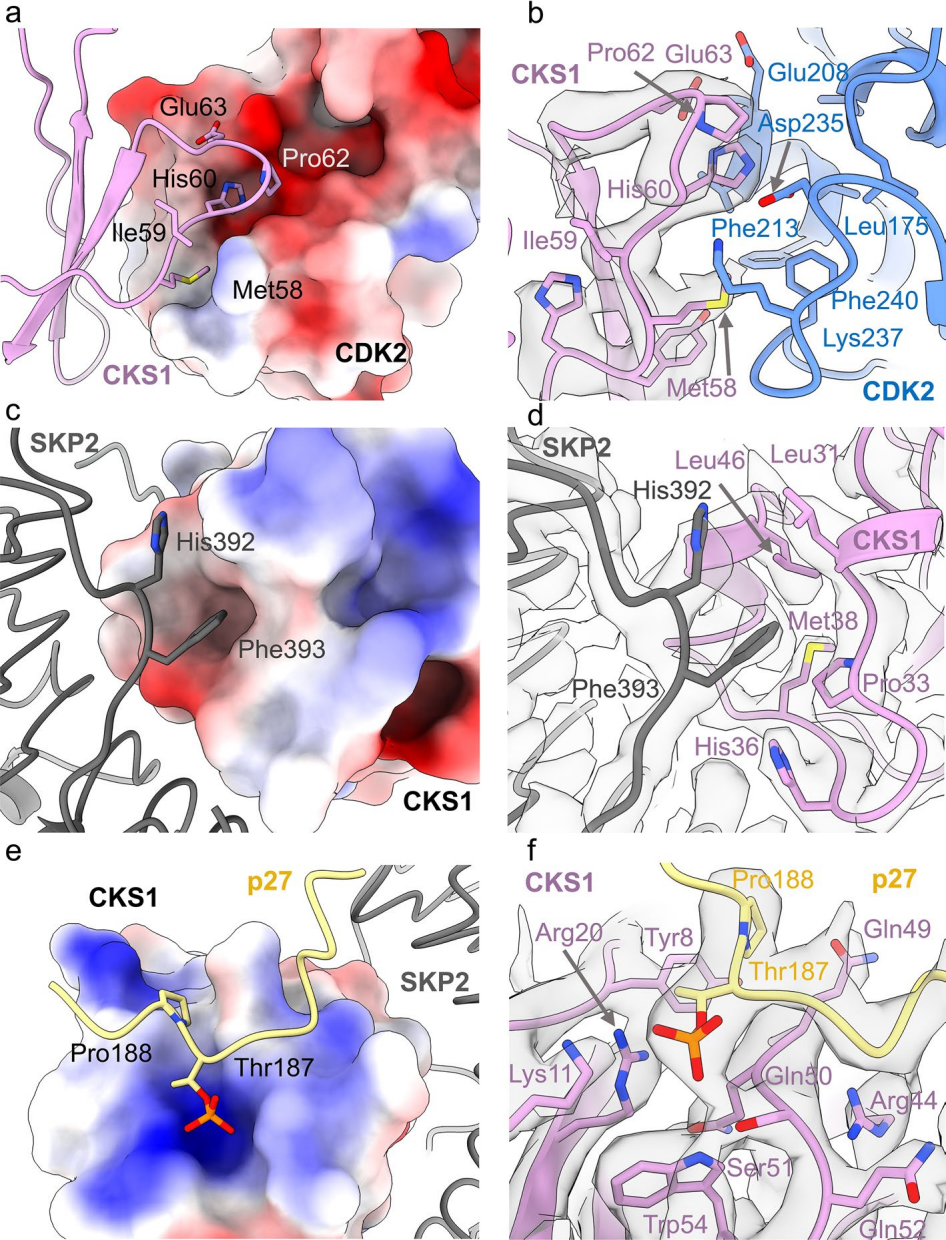
Interaction of CKS1 with CDK2, SKP2 and p27. (**a**,**b**) Interface between CKS1 (pink) and CDK2 (blue). (**a**) Electrostatic surface potential of CDK2 showing the narrow groove in which CKS1 residues Met58-Glu63 bind. (**b**) Ribbon diagram of CDK2-CKS1 interface highlighting the CDK2 residues that form the surface groove (Lys237, Phe240, Phe213, Asp235, Leu175) and density (at 8.8σ, threshold 0.337) for the interacting residues of CKS1, notably His60 and Pro62. (**c**,**d**) Interface between SKP2 (grey) and CKS1 (pink). (**c**) Electrostatic surface potential of CKS1 depicting the hydrophobic pocket in which SKP2 Phe393 binds (**d**) Ribbon diagram of SKP2-CKS1 interface showing density (at 8.8σ, threshold 0.406) for the binding of SKP2 Phe393 and His392 (**e**,**f**) Binding of p27 (yellow) with CKS1 (pink). (**e**) Electrostatic surface potential of CKS1 revealing the binding of p27 phosphorylated Thr187 in a positively charged pocket and Pro188 in a hydrophobic groove (**f**) Ribbon diagram of p27 showing density (at 8.8σ, threshold 0.346) for the phosphorylated Thr187 protruding into CKS1 whilst p27 Pro188 stacks over CSK1 Tyr8 and is wedged by CKS1 Gln5 and Gln49.

The local resolution of the complex ranges 3–6 Å, with the highest resolution (~ 3 Å) observed in the LRR of SKP2 and the CKS1 junction (Fig. 1c). In contrast, the poorest resolution (~ 5–6 Å) is observed within SKP1. SKP1 has previously been crystallised in two conformations (compare PDB **1FQV** and **1FS2**^49^) and shows dynamic behaviour which appears important to its function^50^. Such flexibility will likely be preserved in the cryo-EM analysis, therefore, the lower resolution reported for this domain is unsurprising. The outer region of domain 1 (D1, residues 26–37) and the linker helix domain (residues 38–59) of the p27 KID is also reported at a resolution of ~ 5 Å (Fig. 1b,c). This region of p27 binds to cyclin A but is known to be flexible^51,52^. Despite this flexibility, the resolution was sufficient to model the helical backbone of the KID wrapping around cyclin A and engaging with the RxL binding motif (Fig. 1b). The resolution of p27 improves to ~ 4 Å across domain 2 (D2, residues 60–88) as it extends towards the Thr160-phosphorylated activation segment of CDK2 (residues 145–172, between DFG and APE motifs) (Fig. 1b,c). In accordance with the comparatively higher resolution of CKS1, the bound C-terminal domain of p27 (residues 181–190) was modelled to an improved resolution of ~ 3.5–4.0 Å (Fig. 1c), revealing density for Thr187 phosphorylation (discussed below and illustrated in Fig. 2e,f).

The resolution of this hexameric reconstruction permitted structural analysis of the individual proteins within the full complex using existing crystal structures of CDK2-cyclinA-p27 (PDB **1JSU**^39^) and SKP1-SKP2-CKS1-p27 (PDB **2AST**^38^) as starting models. Whilst these crystal structures can be overlaid to generate a predicted model, the cryo-EM complex presented here provides the first experimentally determined structure of the isolated CDK2-cyclin A-CKS1-p27-SKP1-SKP2 complex. This structure serves as a comparison with the octameric CUL1-RBX1-SKP1-SKP2-CKSHS1-CyclinA-CDK2-p27 complex (PDB **7B5R**^48^) which was recently solved by cryo-EM, not from the isolated octameric complex but following focussed refinement of the SCF-RBR E3-E3 super-assembly complex (PDB **7B5L**^48^).

### CKS1 binds CDK2, SKP2 and p27

The interaction between CDK2 and CKS1 is entirely consistent with the CKS1-CDK2 crystal structure^53^ (PDB **1BUH**), in which CKS1 interacts exclusively with the C-terminal lobe of CDK2. This interaction is underpinned by one loop of CKS1 (residues 58–64), which protrudes into a groove on the surface of CDK2 (Fig. 2a,b). At this interface, the side chain of CKS1 Glu63 hydrogen bonds with the backbone of CDK2 Glu208, whilst the backbone of CKS1 Ile59 forms a hydrogen bond to the side chain of CDK2 Lys23.

CKS1 also associates with the LRR of SKP2, and the resolution of the EM map in this region was sufficient to model side chains and validate the interactions against the SKP1-SKP2-CKS1-p27 crystal structure (PDB **2AST**^38^). At the SKP2-CKS1 interface, a cluster of CKS1 residues form a hydrophobic pocket in which SKP2 Phe393 and His392 bind to stabilize the C-terminal loop (Fig. 2c,d). We previously demonstrated the importance of Phe393 in facilitating CKS1 binding by mutagenizing this residue to a Gly-Ser pair which compromised the interaction^46^.

CKS1 also binds the C-terminal phospho-peptide of p27. Unfortunately, the flexible nature of p27 prevented modelling of the full sequence, however, C-terminal residues 181–190 including the phosphorylated Thr187 residue were solved to 3.5–4.0 Å. This C-terminal peptide lies over the SKP2-CKS1 junction, where the phosphorylated Thr187 sidechain protrudes into a small, positively charged pocket formed by CKS1 Tyr8, Lys11, Arg20, Ser51, Trp54 and Arg71 (Fig. 2e,f). Analysis of the CKS1 surface hydrophobicity profile also identifies a role for p27 Pro188, which stacks on top of CKS1 Tyr8 and is wedged by CKS1 Gln5 and Gln49 to provide a linkage to CKS1 whilst constraining p27 conformation through its intrinsic peptide angles (Fig. 2e,f). Whilst PDB **2AST** reports the p27 C-terminal peptide lying flat over the SKP2-CKS1 junction, our density suggests a more condensed, potentially helical conformation**.**

### Engagement of the p27 KID with cyclin A and CDK2

**Figure 3:**
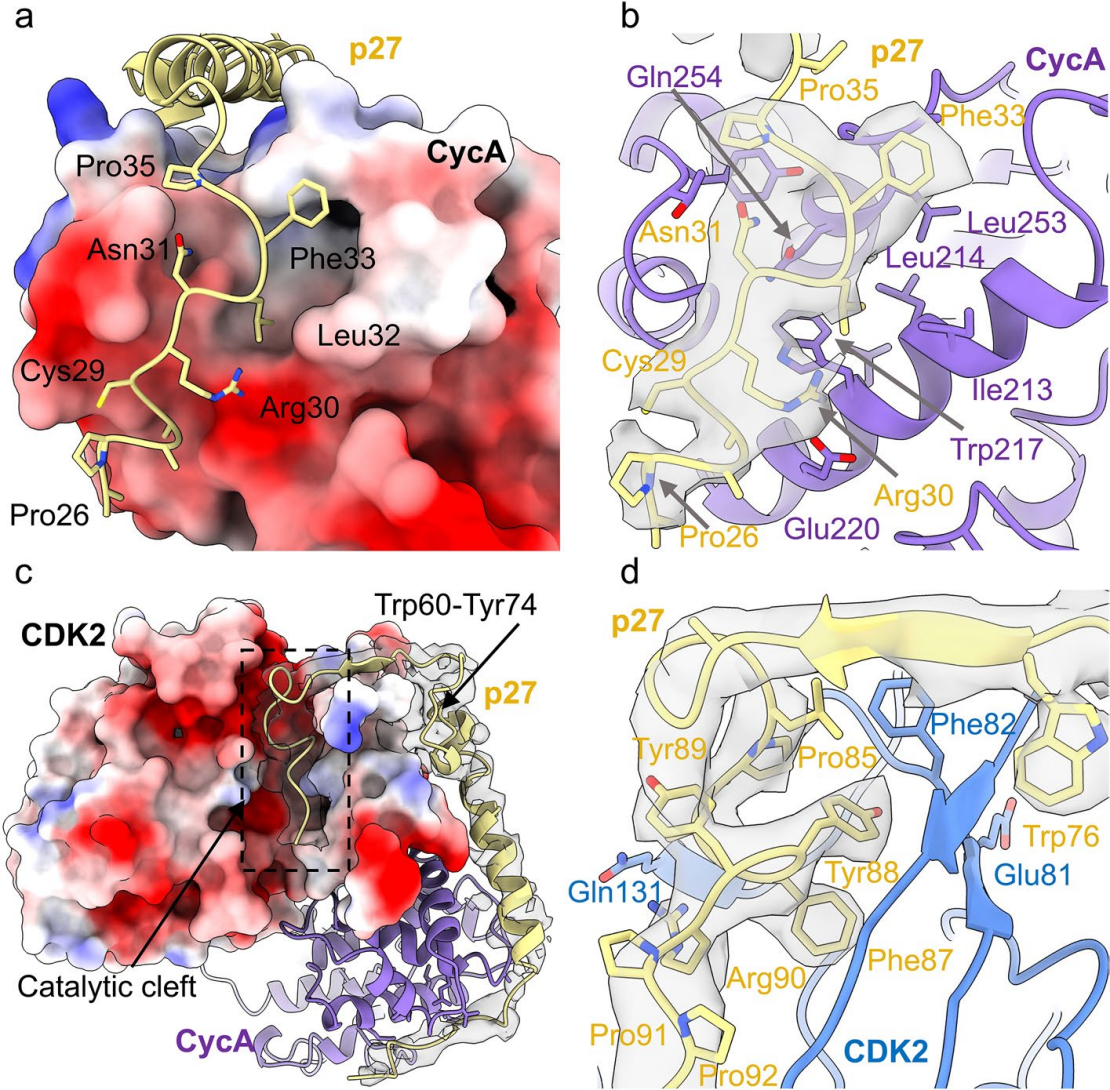
Binding of p27 with cyclin A and CDK2. (**a**,**b**) Interaction of p27 (yellow) with cyclin A (purple). (**a**) Electrostatic surface potential of cyclin A revealing binding of the p27 N-terminus in a shallow groove along the cyclin box repeat. The conserved p27 Leu-Phe-Gly sequence binds in a deeper, more hydrophobic groove. (**b**) Ribbon and density (at 6.0σ, threshold 0.205) diagram of p27 binding to the ⍺-helices of the cyclin box repeat of cyclin A, where residues Ala28, Arg30 and Asn31 of p27 form hydrogen bonds with Trp127, Glu220 and Gln254 of cyclin A respectively. (**c**,**d**) Binding of p27 (yellow) with CDK2 (blue). (**c**) Electrostatic surface potential of CDK2 showing how p27 (density at 6.0σ, threshold 0.221) extends over CDK2 into the catalytic cleft, with residues Trp60, Phe64, Phe62, Tyr74 and Trp76 of p27 interacting with CDK2 (**d**) p27 3_10_ helix (residues 85–93) binds through the catalytic cleft of CDK2 and into the ATP binding site where p27 Tyr88 hydrogen bonds with the backbone of CDK2 Glu81 (density map at 8.8σ, threshold 0.315).

Analysis of the p27 KID (residues 25–59) allowed us to model p27 engaging with the RxL binding motif of cyclin A and follow its path towards the CDK2 activation segment, as reported in the CDK2-cyclinA-p27 crystal structure (PDB **1JSU**^39^). The N-terminal coil of p27 (D1), containing the CIP/KIP conserved Leu32-Phe33-Gly34 (LFG) sequence starts at Pro26 and is rigidified by intramolecular hydrogen bonds between Cys29 and Pro26, and between Ser27 and Arg30. This rigid coil binds in a shallow groove on the surface of the cyclin box fold (N-CBF) (Fig. 3a) and is stabilised by various hydrogen bonds; p27 Ala28, Arg30 and Asn31 hydrogen bond with cyclin A Trp218, Glu220 and Gln254 respectively. The conserved LFG sequence sits in a deeper, more hydrophobic groove formed by cyclin A Ile213, Leu214, Trp217, Arg250, Leu253 and Gly251, (Fig. 3a,b), which also constitute van der Waals contacts with the p27 Leu-Phe pair. Following the LFG sequence, p27 forms an α-helix which rests over the α5-helix of cyclin A (residues 288–301) and extends towards CDK2 (Fig. 3a,c) (PDB **1JSU**).

Consistent with PDB **1JSU**, we observe the β-hairpin of p27 (residues 60–71) packing against the N-terminal β-sheet of CDK2 to bury a cluster of aromatic residues, namely the conserved CIP/KIP residues Trp60, Phe64, Phe62, Tyr74 and Trp76 (Fig. 3c). Additionally, CDK2 Tyr77 hydrogen bonds with p27 Phe64 to stabilise the interaction. At the second region of the p27-CDK2 interface, the p27 β-strand (residues 75–81) forms a hybrid β-sheet with CDK2 (Fig. 3d), creating an interface that is dictated by hydrogen bonds between p27 Gln77 and CDK2 Lys20, and p27 Val79 and CDK2 Val18. Consistent with the crystal structure PDB **1JSU**, we were unable to model the first β-strand of CDK2 (residues 1–13), suggesting it is disordered in solution upon p27 binding. The final component of the p27-CDK2 interaction involves the p27 3_10_ helix (residues 85–93), which binds through the catalytic cleft of CDK2 into the ATP binding site (Fig. 3d). This interaction is well conserved with the crystal structure (PDB **1JSU**) and highlights that p27 Tyr88 secures the binding by forming hydrogen bonds with CDK2 Glu81 and Leu83, and making van der Waals contacts with CDK2 Phe82 and Leu83 (Fig. 3d). Additionally, we were able to identify other key p27 residues; Phe87 sits deep in the active site cleft wedged by CDK2 residues Lys33, Leu134, Ala144 and Phe80, whilst p27 Arg90 hydrogen bonds with CDK2 Gln131 (Fig. 3d).

**Figure 4:**
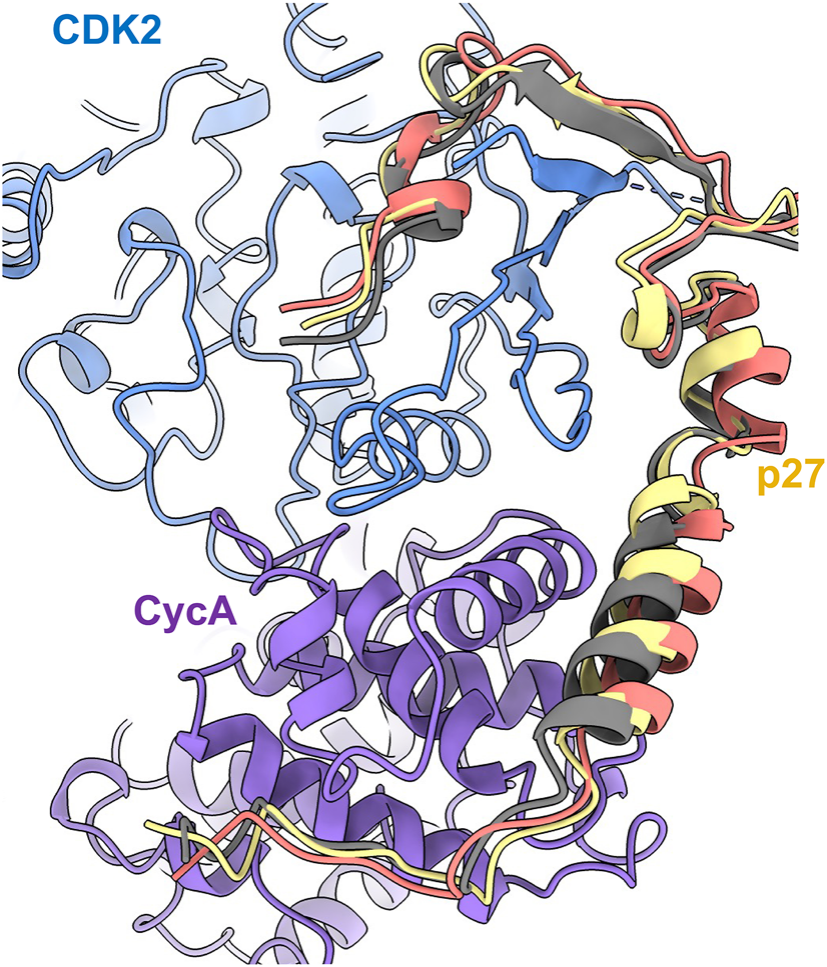
Helical conformation of p27 KID bound to CDK2-cyclin A. The CDK2, cyclin A and p27 subunits of the hexameric complex are shown in blue, purple and yellow respectively. The CDK2-cyclin A modules of CDK2-cyclin A-p27 (PDB **1JSU** (grey)) and the octameric SCF^SKP2^ E3 ligase structure (PDB **7B5R** (salmon)) were superposed with the hexameric CDK2-cyclin A module, and the p27 domains rendered in grey (PDB **1JSU**), salmon (PDB **7B5R**) and yellow (hexameric complex) respectively, to reveal different helical conformations for p27 KID when bound to CDK2-cyclin A.

In general, the binding of p27 in the hexameric complex is broadly consistent with PDB **1JSU** and PDB **7B5R**. However, all structures exhibit varying helical conformations of the KID as it extends towards the N-terminal lobe of CDK2 (Fig. 4). This supports analysis by Lacy and colleagues^52^ in which p27 was found to be dynamic, transitioning from disordered to nascent secondary structure on target binding, which may have implications for molecular recognition.

### 3D variability analysis reveals hinge motion within the hexameric complex

**Figure 5:**
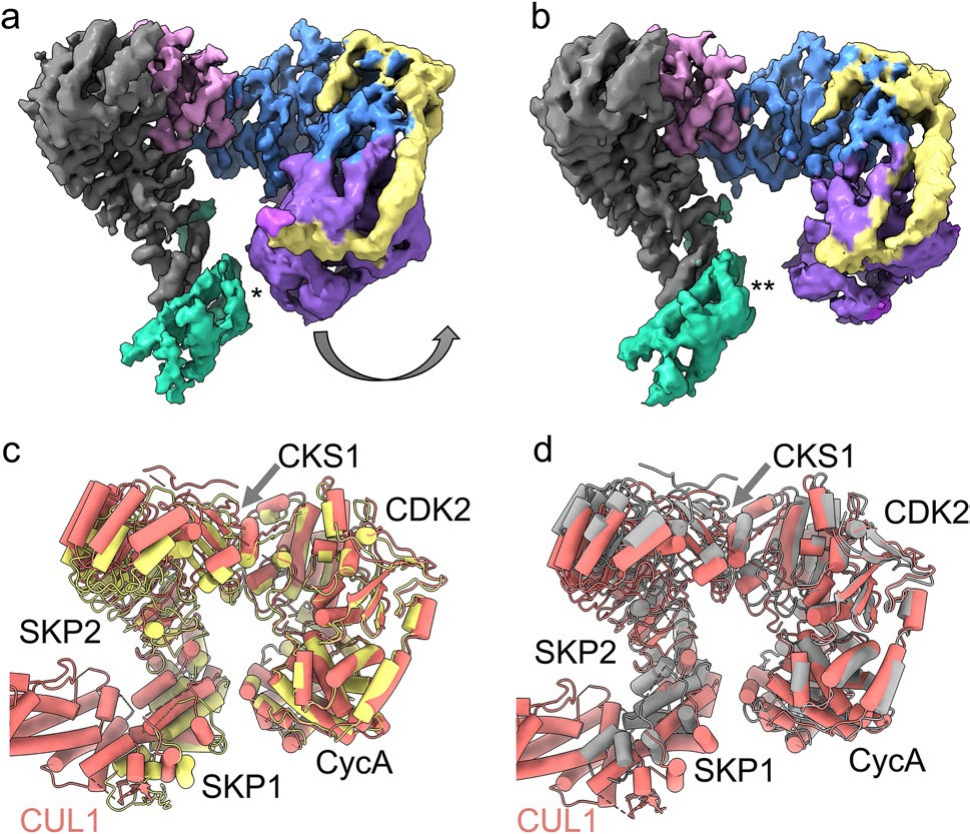
3D variability analysis of the hexameric complex. (**a**,**b**) Cryo-EM maps representing the (**a**) closed conformation and (**b**) open conformation of the hexameric complex as revealed by 3D variability analysis. Cyclin A (purple) bound to p27 (yellow) swings away from N-terminal SKP2 (grey) and SKP1 (green) motif whilst the SKP1 domain concomitantly extends away from the cyclin A-p27 unit. Average distance between SKP1 and cyclin A is ~ 8 Å in closed conformation (as indicated by *) and ~ 16 Å in open conformation (as indicated by **). (**c**,**d**) Comparison of the octameric complex PDB **7B5R** with the open and closed conformations of the hexameric complex. Superposition of PDB **7B5R** (salmon) with the (**a**) closed conformation (yellow) and (**b**) open conformation (grey) of the hexameric complex showing that **7B5R** falls within the broad conformational space of the hexameric complex. The average distance between SKP1 and cyclin A is ~ 10 Å in PDB **7B5R**.

Given the inherent flexibility of the constituent components, we utilised 3D variability analysis^54^ to visualise the conformational space of the hexameric complex. This analysis revealed a “breathing” motion in which p27 bound to cyclin A moves inwards and outwards from the SKP2 and SKP1 domains using CKS1 as a hinge point (Fig. 5, Supplementary: hexamer.mov S1). In the closed conformation the average distance between cyclin A and SKP1 is ~ 8 Å, whilst in the open conformation the average distance is ~ 16 Å (Fig. 5a,b). This motion is also reflected in the p27 KID, which concertedly moves with cyclin A as the complex transitions from closed to open, further supporting its flexibility. Although less significant, some motion was also identified in SKP1, which concomitantly extends away from cyclin A.

On comparison, it is evident that the octameric SCF^SKP2^ E3 ligase structure (PDB **7B5R**) falls within the broad conformational space of the hexamer (Fig. 5c,d), suggesting the complex is generally unperturbed by CUL1. Whilst the octameric complex does not align directly with the open or closed conformation, it is most consistent with the closed form, with an average distance of ~ 10 Å between SKP1 and cyclin A (Fig. 5c,d). This analysis suggests a model in which p27 binding to CDK2-cyclin A-CKS1 generates a flexible substrate which, as it transitions between open and closed conformations, can be captured in a conformation that promotes recognition by SCF^SKP2^. This motion is typical of the large structural rearrangements often required to bring the E2 and substrate together within the cullin-RING ligase complex^55^. In the case of p27, the SKP2 substrate recognition motif is the limiting component in ubiquitin-mediated degradation^27^. We speculate the observed motion may facilitate p27 recognition by bringing the substrate towards the SKP2 recognition component. This motion may also enhance p27 ubiquitination by shortening the E2-substrate distance for initiation of ubiquitination in the closed conformation; or in the open form, increase the space at the heart of the complex to assist the catalytic cycle. However, this study is limited in its biochemical scope to elucidate the functional importance of this motion beyond speculation. Future efforts could focus on evaluating the conformational space of the pentameric CDK2-cyclin A-CKS1-SKP1-SKP2 complex to determine if this flexibility profile exists in the absence of p27.

### Particle subtraction identifies further features of the hexameric complex

In light of the 3D variability analysis, we performed particle subtraction and local refinement of the SKP1-SKP2-CKS1 (PDB **8BYL**, EMD-**16327**) and CDK2-cyclin A-p27 (PDB **8BZO**, EMD-**16344**) portions of the complex in an effort to improve the local resolution (Supplementary Fig. S5).

**Figure 6:**
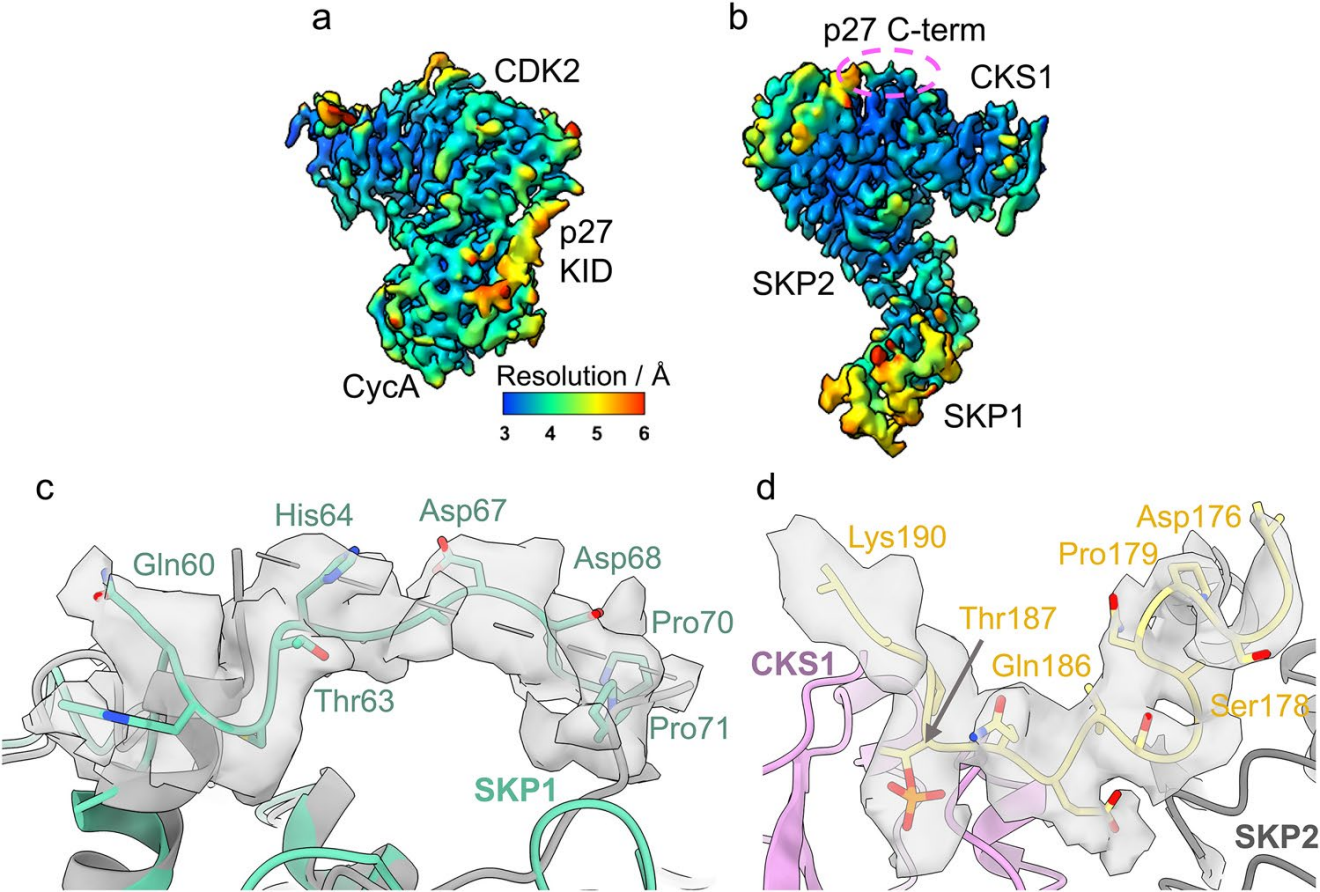
Local resolution analysis of particle subtraction maps. (**a**,**b**) Local resolution analysis of (**a**) particle subtraction map of CDK2-cyclin A-p27 (PDB **8BZO**, EMD-**16344**), showing enhanced resolution of the CDK2-cyclin A core and the KID of p27. (**b**) Particle subtraction map of SKP1-SKP2-CKS1 (EMD-**16327**, PDB **8BYL**) demonstrating improved resolution in the SKP1 domain and the phosphorylated C-terminus of p27 bound to CKS1 (circled in pink). (**c**) Particle subtraction and local refinement of SKP1-SKP2-CKS1, density (depicted at 4.0σ, threshold 0.110) yields an improvement in map continuity and resolution for SKP1 loop 2 which was not observed in the full hexameric model (grey, as indicated by dotted linker). (**d**) Improvement of density (at 3.2σ, threshold 0.143) for the C-terminus of p27 bound over CKS1 allows previously unobserved residues Asp176, Gly177, Ser178, Pro179 and Asn180 to be modelled extending towards the LRR of SKP2.

Following subtraction of the SKP1-SKP2-CKS1 signal and local refinement of CDK2-cyclinA-p27, the resolution of cyclin A was improved from 4.5–5.0 Å to 3.5–4.0 Å (compare Fig. 1c and Fig. 6a). Concomitant improvements throughout the p27 KID (Fig. 6a) were also observed, permitting modelling of side chains that previously could not be discerned (Supplementary Fig. S6a,b). Using the opposing strategy of subtracting signal for CDK2-cyclin A-p27 and refining SKP1-SKP2-CKS1 (PDB **8BYL**, EMD-**16327**), the resolution of CKS1, the bound p27 C-terminus, and the LRR domain and N-terminus of SKP2 were all improved (compare Fig. 1c with Fig. 6b). The most drastic improvement was observed in SKP1, in which the resolution was enhanced from 5–6 Å to 3.5–5.5 Å (Fig. 6b), allowing previously unmodelled residues in loop 2 (residues 64–71) (Fig. 6c) and loop 1 (residues 31–41) to be observed. However, the full length of loop 1 could not be modelled and comparison with the octameric complex (PDB **7B5R**) suggests this region of SKP1 forms part of the interface with CUL1 and may rigidify on CUL1 binding.

Local refinement of SKP1-SKP2-CKS1 provided opportunities for further modelling in SKP2 and C-terminus of p27. In the full hexameric map, the first observable SKP2 residue was Val95, whilst Pro93 and Gly94 could be modelled in the particle subtraction map, providing additional N-terminal residues not reported in the crystal structure (PDB **2AST**). This marginally improved N-terminal coverage on local refinement lends some weight to the N-terminus of SKP2 being disordered in the absence of cyclin A binding. Owing to the enhanced resolution of the SKP2-CKS1 junction, density for the bound p27 C-term peptide was also improved to a resolution ~ 3.0–3.5 Å (Fig. 6b). This permitted backbone modelling of additional residues (Gly177, Ser178, Pro179 and Asn180) that extend back towards the LRR of SKP2 (Fig. 6d) with p27 Gly182 protruding over SKP2. An overlay with structures PDB **2AST** and **7B5R** suggests some divergence in the conformation of this p27 peptide, however, our particle subtraction map suggests a condensed, potential helical conformation (Supplementary Fig. S7).

Using cryo-EM, we provide an in-depth structural analysis of the quaternary architecture of the CDK2-cyclin A-CKS1-SKP1-SKP2-p27 complex which forms part of the SCF^SKP2^ E3 ligase that controls p27 ubiquitin-mediated degradation. Our experimentally determined structure supports existing crystal structures of CDK2-cyclin A-p27^39^ and SKP1-SKP2-CDK1-p27 peptide^40^ complexes, whilst utilising the power of cryo-EM to highlight previously unidentified dynamic behaviour. Through 3D-variability analysis, we report significant structural flexibility that gives rise to open and closed conformations of the hexameric complex. It remains to be determined whether the hexamer retains this flexibility upon binding of CUL1 and RBX1, or whether incorporation into the octameric complex or the SCF-RBR E3-E3 super-assembly selects compatible hexamer conformational states. From either model it can be hypothesized that the ability of CKS1 to act as a hinge point may promote p27 regulation by SCF^SKP2^ through facilitating p27 recognition and ubiquitination. More broadly, we demonstrate the influence that the combined use of 3D-variability analysis and local refinement can have on particle clarity, which may be widely applicable to flexible protein complexes.

## Materials and methods

### Protein expression

Full-length human CDK2 was co-expressed with *S. cerevisiae* GST-CAK1 from a pGEX-6P-1 vector as a GST-fusion protein cleavable with 3C protease as described^56^. The construct yields a CDK2 sequence preceded by Gly-Pro-Leu-Gly-Ser and is phosphorylated on Thr160 (T160pCDK2). Untagged human cyclin A2 (residues 174–432)^56^ and full-length human His_6_-CKS1^57^ were expressed using the pET21d vector, and full-length GST-p27 (residues 1–198) was expressed from a modified pGEX-6P1 vector^14^. Full-length GST-SKP2 (3C cleavable) was expressed with full-length SKP1 from a modified pET3d backbone^46^. The cloning strategy leaves Gly-Pro at the start of SKP2. All constructs were expressed in BL21-DE3 (pLYS-S) cells grown at 30 °C in LB media till OD_600_ ~ 0.7–0.8, induced with 0.2 mM IPTG and incubated for 16 h at 18 °C. Cell pellets were resuspended in HEPES buffered saline (mHBS, 20 mM Tris pH 7.6, 300 mM NaCl, 1 mM DTT) with 200 μM RNAase A, 200 μM DNAaseI, lysed by sonication and clarified by centrifugation.

### Protein purification

The SKP1-SKP2 complex and p27 were purified by affinity chromatography on a glutathione-sepharose column (Merck) equilibrated in mHBS as described previously^46^. Proteins were eluted by 20 mM glutathione in mHBS pH 8.0, cleaved with 3C protease (1:50 w/w) and further purified using size exclusion chromatography (SEC) (Superdex 200 HR 16/60 (Cytiva)) equilibrated in 20 mM Tris pH 7.8, 300 mM NaCl, 0.5 mM TCEP. T160pCDK2-cyclin A complex was prepared as previously described^56^. Briefly, a glutathione-sepharose column equilibrated in mHBS was charged with GST-T160pCDK2, washed to baseline and then used as an affinity column to purify untagged cyclin A. After washing to baseline T160pCDK2-cyclin A was eluted with 20 mM glutathione in mHBS, cleaved with 3C protease and subjected to SEC (Superdex 200 HR 16/60 (Cytiva)) equilibrated in 20 mM Tris pH 7.8, 300 mM NaCl, 0.5 mM TCEP. Co-eluting GST dimer was removed through subtractive GST. CKS1 was purified by affinity chromatography on an HiTrap His column (Cytiva) equilibrated in 40 mM HEPES pH 7.0, 200 mM NaCl, 25 mM imidazole and eluted with a gradient of 300 mM imidazole before loading onto a Superdex75 column (Cytiva) equilibrated in 20 mM Tris pH 7.8, 300 mM NaCl, 0.5 mM TCEP. The His6 tag was not cleaved.

### Phosphorylation of p27 T187 and hexameric assembly

T160pCDK2-cyclinA-p27 (5.3 mg) in 40 mM Tris–HCl (pH 7.6), 1 mM ATP, 10 mM MgCl_2_ buffer was incubated with 4.2 mg of T160pCDK2-cyclin A in a total volume of 2 mL at 25 °C for 2 h. The reaction mix was loaded on to a Superdex 200 16/60 HR column (Cytiva) equilibrated in 20 mM Tris pH 7.8, 300 mM NaCl, 0.5 mM TCEP buffer to remove the T160pCDK2-cyclin A. To assemble the complex, equimolar concentrations of purified CDK2-cyclinA-p27 (phosphorylated on CDK2 T160 and p27 T187) and SKP1-SKP2 complexes were incubated with a molar excess of CKS1 for 1 h at 4 °C and separated by SEC on a Superdex 200 10/300 column (Cytiva) equilibrated in 20 mM Tris pH 7.8, 300 mM NaCl, 0.5 mM TCEP.

### Data collection

Particle distribution was assessed by analysis of negatively stained samples using uranyl acetate on a 120 kV microscope (Hitachi TS8700). The complex was subsequently loaded on 400-mesh UltrAuFoil R1.2/1.3 (Quantifoil) grids cleaned using Tergeo-EM plasma cleaner (PIE Scientific) for 1 min in remote plasma generated by an RF power (W) of 15, pulse ratio of 255, using a mixture of nitrogen, oxygen and argon. 3 μl of hexameric complex (0.2 mg/ml) were blotted on the grids for 6 s with a blot force of -6 using a Vitrobot MK IV (Thermo Fisher Scientific) operated at 95% humidity at 5 °C. The grids were vitrified in liquid ethane and screened on a Titan Krios microscope (Thermo Fisher Scientific) operating at 300 kV with an energy-filtered K2 direct detector with an energy filter set with a 20 eV slit width (Gatan). Data acquisition was setup using EPU software (Thermo Fisher Scientific). 762 electron movies were recorded at 130 k magnification in counting mode with a pixel size of 1.07 Å and a total dose of 65.8 e/Å^2^. Data were collected at − 1.0 to − 3.0 μm defocus.

### Hexameric complex data processing

All data were processed in CryoSPARC^58^ following the workflow outlined in Supplementary Fig. S2.

### Micrograph correction, autopicking and map generation

762 movies were motion corrected using Patch Motion Correct Multi and the contrast transfer function (CTF) parameters were estimated using Patch CTF Correction Multi. Exposures were curated to remove poor micrographs and the remaining 673 micrographs were used for particle picking using blob picker with a minimum and maximum particle diameter of 70 Å and 160 Å. 1,110,356 particles were picked, extracted with a box size of 300 px (Fourier cropped to 150 px) and 2D classified. After discarding poor particles, 438,448 particles were used to generate 2D templates low pass filtered to 20 Å for template picking.

613,930 template picked particles were subjected to two rounds of 2D classification and the remaining 246,303 particles were used to generate a single ab-initio model that was refined through the homogenous refinement. 50 × 2D templates were generated from the refined map and used for template picking of 669,988 particles. These particles were extracted with a box size of 300 px (Fourier cropped to 150 px) and classified through 3 × 2D classification to yield 217,628 particles. These particles were re-extracted without Fourier cropping and 3D classified using heterogenous refinement. The particles were sorted into 3 × 3D classes, yielding class 2 with best map of 136,325 particles (5.14 Å). This class was refined through homogenous refinement and non-uniform refinement^59^ to yield the final map at 3.4 Å resolution (FSC cut off 0.143). The final map was sharpened using a B-factor of – 121 Å^2^, as indicated by the Guinier plot, and local resolution was estimated.

### 3D variability analysis, particle subtraction and local refinement

3D variability analysis^54^ was performed on the final 136,325 particle set, using a mask that encompassed the full complex, solving for 3 orthogonal principal modes over 20 iterations with a 5 Å resolution filter. The first and last frames were captured to depict the closed and open conformations of the hexamer respectively.

Using the full hexameric map, a mask for the SKP1-SKP2-CKS1 portion of the complex was generated in UCSF ChimeraX^41,42^ and low pass filtered to 20 Å with additional dilation (radius of 5) and padding (soft padding width 16) in CryoSPARC via the volume tools function. This mask was used to subtract signal of the SKP1-SKP2-CKS1 from the particles. Signal subtracted particles were refined using local refinement with a mask (low pass filter 20 Å, dilation radius 5 and soft padding width 16) generated from the CDK2-cyclinA-p27 portion of the complex. The reverse processing of subtracting CDK2-cyclinA-p27 signal and refining the SKP1-SKP2-CKS1 portion was performed to yield two subcomplex maps.

### Model docking, refinement and validation

Initial rigid body docking of subdomains of the hexameric complex was performed in UCSF ChimeraX^41,42^ using CDK2-cyclinA-p27 (PDB **1JSU**) SKP1-SKP2-CKS1-p27 (PDB **2AST**). The maps were imported into Phenix^60^ for subsequent model refinement and validation^61,62^. The fitting of this model was improved with Real-space refinement^43^ followed by manual refinement in COOT^44^ based on the MolProbity^61^ outlines. The model quality was assessed in Phenix using Comprehensive Validation^62^ before deposition to the Protein Data Bank (PDB) and the Electron Microscopy Data Bank (EMDB).

## Supplementary Information

Supplementary Information.

## Data Availability

The map and model of the hexameric CDK2-cyclin A-CKS1-p27-SKP1-SKP2 complex has been deposited in the Protein Data Bank and the Electron Microscopy Data Bank with accession numbers **8BYA**, and **16325** respectively. The particle subtraction maps and models for SKP1-SKP2-CKS1 and CDK2-Cyclin A-p27 are deposited with accession codes PDB **8BYL**/EMD-**16327** and PDB **8BZO**/EMD-**16344** respectively.

## Abbreviations

CBF: Cyclin-box fold
CDK: Cyclin-dependent kinase
CKI: Cyclin-dependent kinase inhibitor
CKS1: Cyclin-dependent kinases regulatory subunit 1
LRR: Leucine rich repeats
RMSD: Root mean square deviation
SEC: Size exclusion chromatography
SKP: S-phase kinase associated protein
FSC: Fourier shell correlation
CTF: Contrast transfer function
