# Supplementary material for: Cryo-EM structure of SKP1-SKP2-CKS1 in complex with CDK2-cyclin A-p27KIP1
Source: Sci Rep. 2023 Jul 3;13:10718. doi: 10.1038/s41598-023-37609-9 (PMC10318019; doi:10.1038/s41598-023-37609-9)
Supplement: Supplementary file 1 — Supplementary Information. [file 41598_2023_37609_MOESM1_ESM.pdf]

Supplementary Information

Cryo-EM structure of SKP1-SKP2-CKS1 in complex with CDK2-cyclin A-p27KIP1

Rhianna J. Rowland, Richard Heath, Daniel Maskell, Rebecca F. Thompson, Neil A. Ranson, James N. Blaza, Jane A. Endicott, Martin E.M. Noble and Marco Salamina

Running title: Cryo-EM structure of the CDK2-cyclin A-p27KIP1-CKS1-SKP1-SKP2 complex

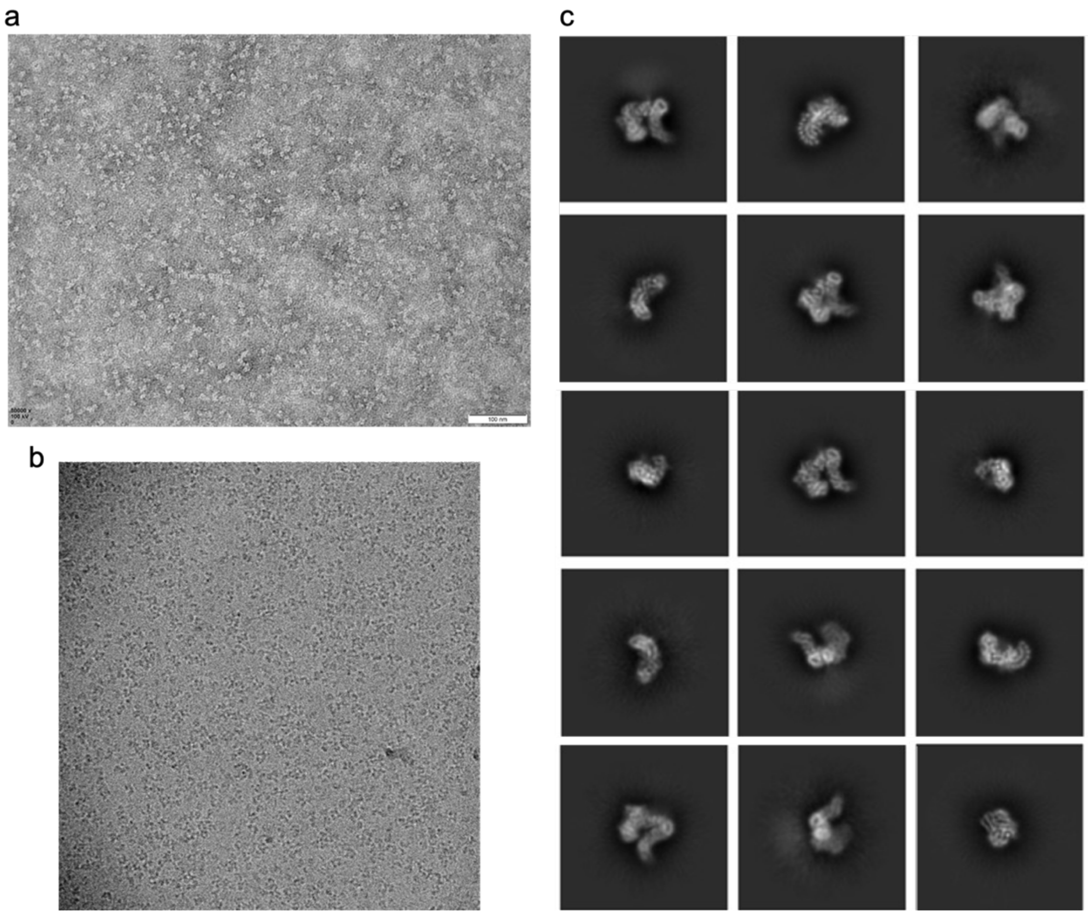

Supplementary Figure S1. Screening of hexameric particles. Related to Figure 1.

(a) Negative stain micrograph of the hexameric complex. (b) Representative single particle cryo-EM micrograph of the hexameric complex from a hole on a UltrAuFoil R1.2/1.3 400 mesh grid. Micrograph is representative of 762 micrographs from a single data collection. (c) 2D class averages of the final refined particles.

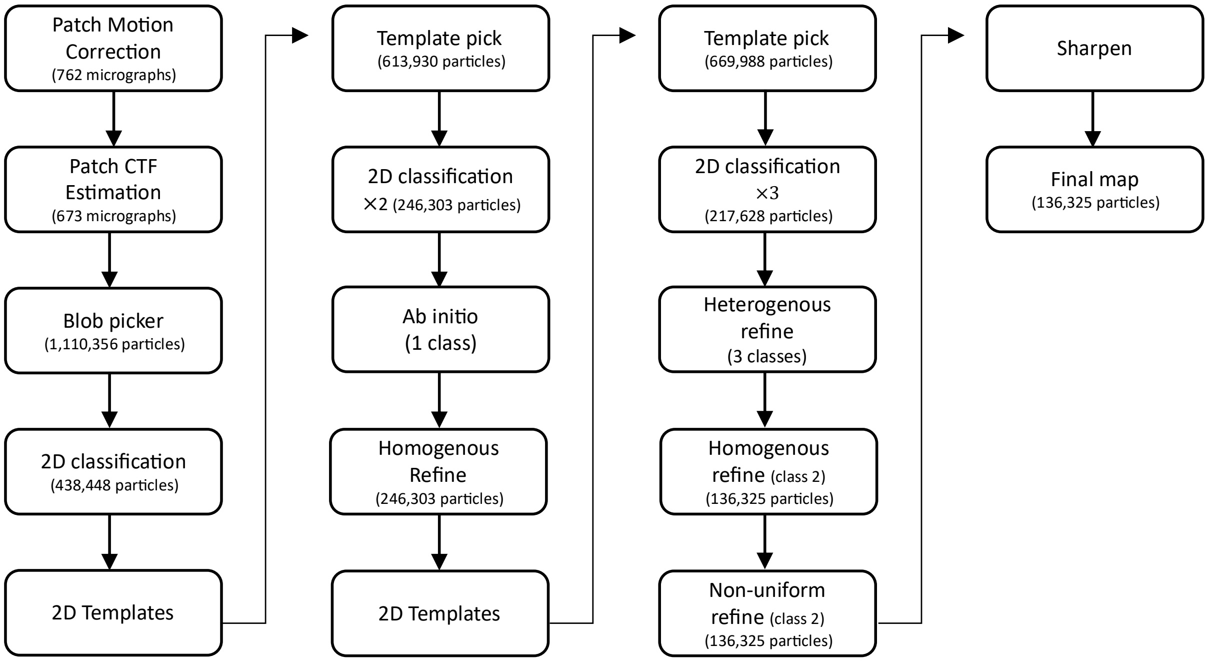

Supplementary Figure S2. Cryo-EM processing to generate the 3.4 Å map of the hexameric complex. Related to Figure 1.

Blob picking of 1,110,356 particles followed by 2D classification yielded 2D templates that were used to pick 613,930 particles. The particles were 2D classified and used to generate an *ab initio* model (246,303 particles) which was refined by homogenous refinement to generate more exhaustive templates. Following further template picking and 2D classification, particles were separated into 3 x 3D classes and the best class (136,325 particles) was refined by homogenous and non-uniform refinement. The map was sharpened (B-factor = -121 Å^2).

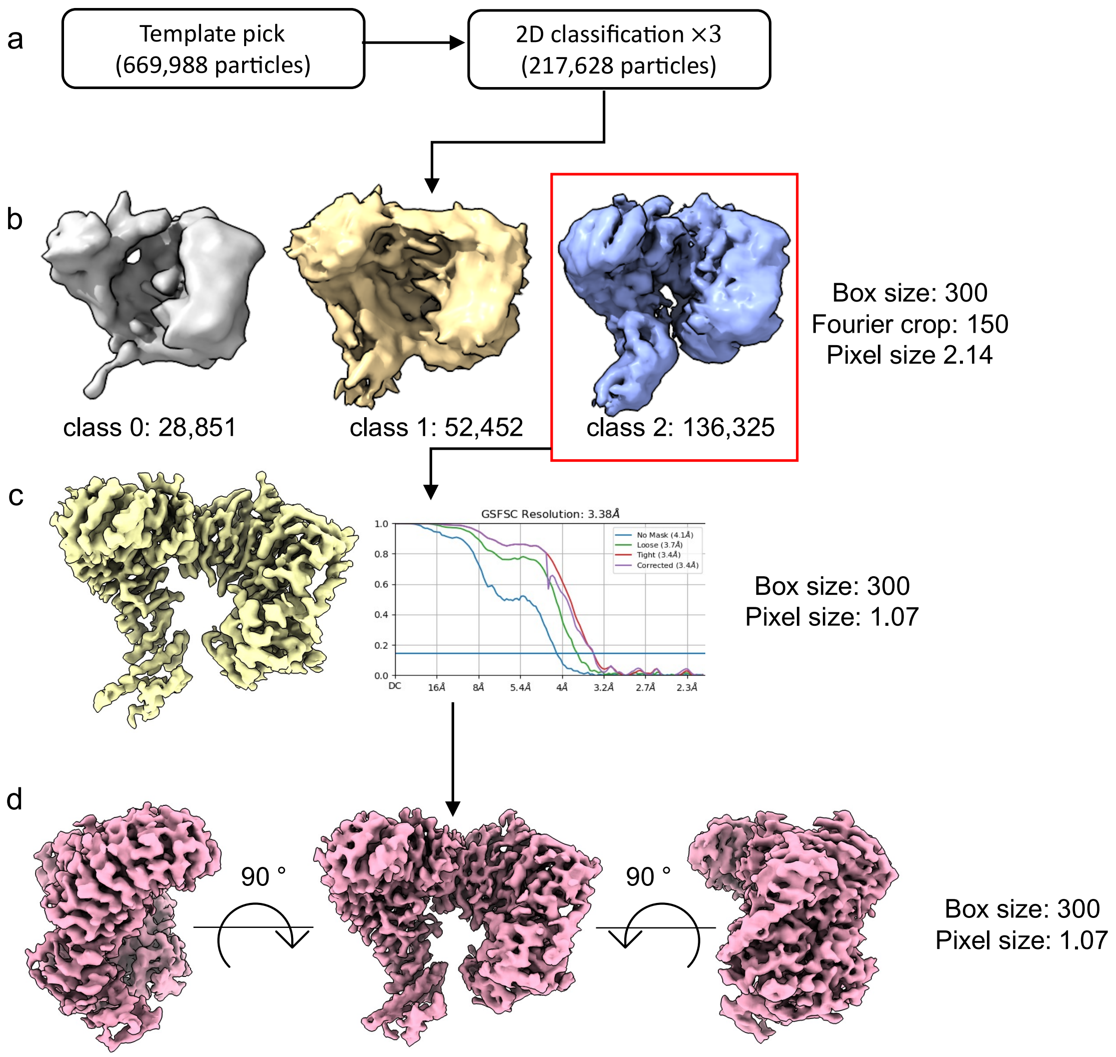

Supplementary Figure S3. Cryo-EM processing of the hexameric CDK2-Cyclin A-p27-CKS1-SKP1-SKP2 complex. Related to Figure 1.

Following template picking and 2D classification (a) 217,628 particles were sorted into (b) 3 × 3D classes through heterogenous refinement (class 0: 28,851 particles class 1: 52,452 particles and class 2: 136,325 particles) (c) Class 2 was refined by homogenous and non-uniform refinement to yield (d) a 3.4 A cryo-EM map of the full hexameric complex.

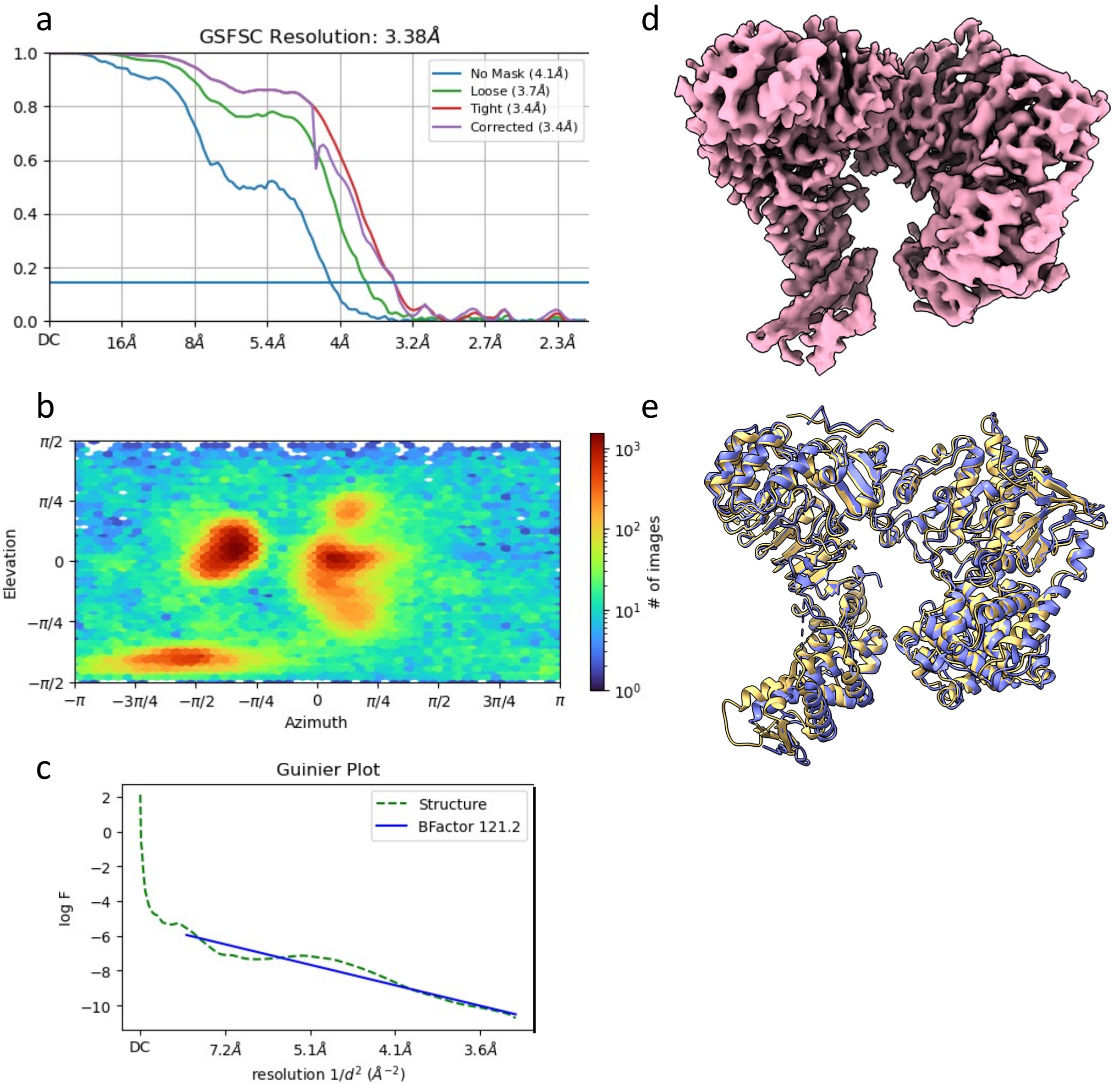

Figure S4: Refinement information for full hexameric complex map. Related to Figure 1.

(a) Gold standard Fourier shell correlation plot for the refined 3.4 Å map. FSC = 0.143. (b) Orientation distribution heat map of the refined particle set (c) Guinier plot of final refinement. Global B-factor = 121 $Å^2$. (d) final cryo-EM map. (e) Overlay of the initial hexameric model generated in ChimeraX (yellow) using PDB **2AST** and PDB **1JSU** as starting models and the final, refined hexameric model (purple) PDB **8BYA,** revealing moderate conformation changes in SKP1 and p27 KID.

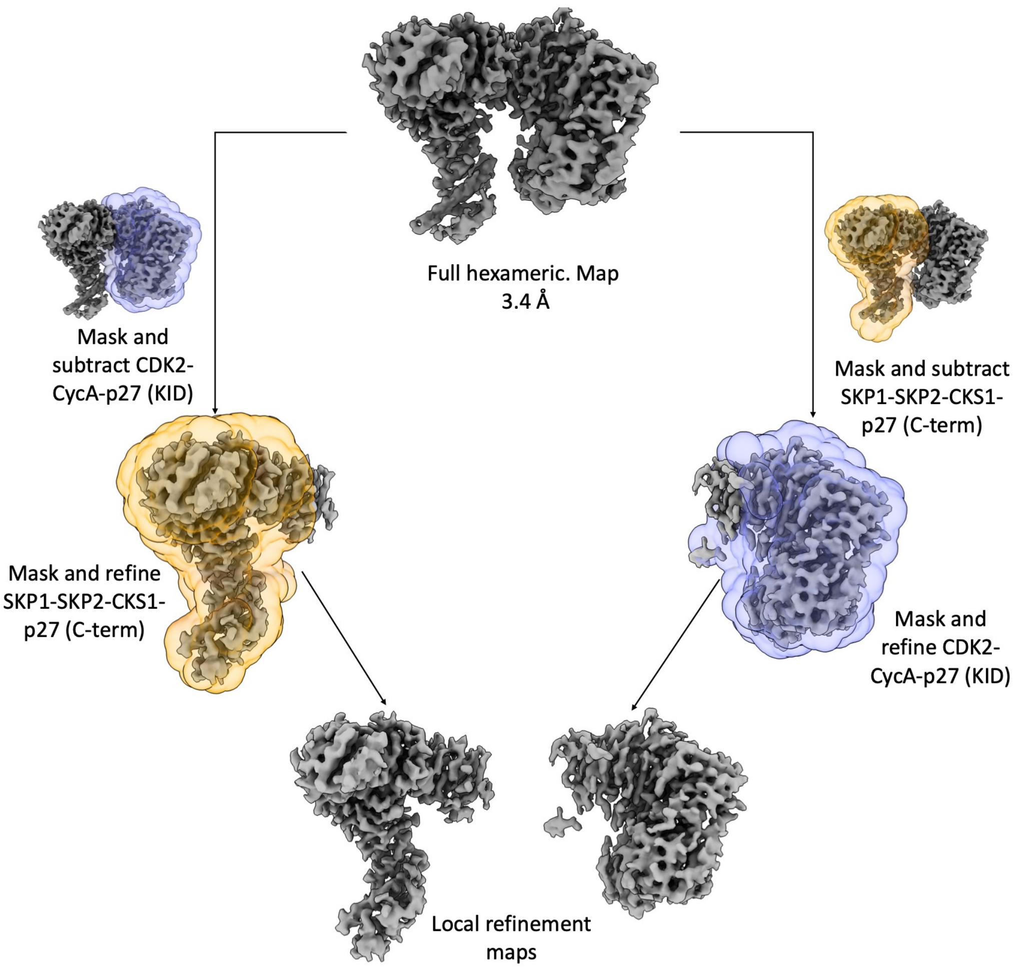

Figure S5: Particle subtraction and local refinement of the hexameric complex. Related to Figure 6.

The full hexameric map was refined in two sub-complexes; a mask (purple) was made to subtract CDK2-Cyclin A-p27(KID) signal and a mask (orange) for SKP1-SKP2-CKS1-p27 (C-term) was used to locally refine this portion of the complex. These masks were subsequently used to perform the reverse process and subtract the SKP1-SKP2-CKS1-p27 (C-term) and locally refine CDK2-Cyclin A-p27(KID) to produce two subcomplex maps that constitute the complete hexameric complex.

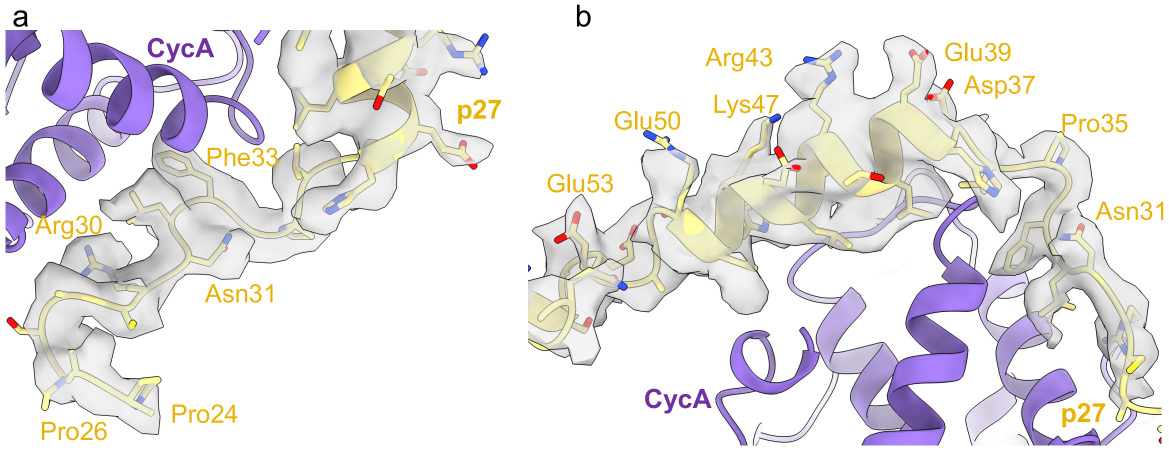

Figure S6: Improved resolution of p27 following particle subtraction and local refinement. Related to Figure 6. Density (at 6.5 s) for p27 (yellow) resulting from particle subtraction and local refinement of CDK2-cyclin A-p27 revealing density for (a) an additional Pro24 residue at the N-terminus (threshold 0.284) and (b) for side chains along the length of the p27 KID that were previously unobserved in the full hexameric assembly (threshold 0.228).

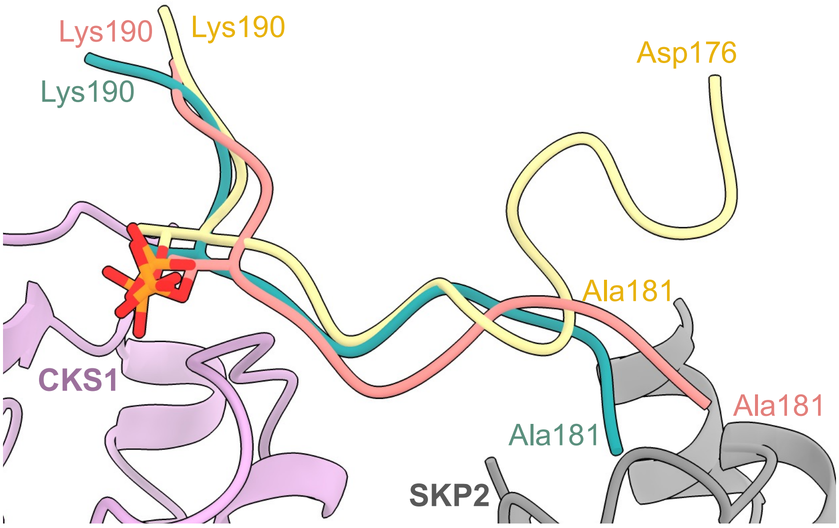

Figure S7: p27 C-terminus bound at the CKS1-SKP2 junction. Related to Figure 6.

Overlap of the p27 C-terminus modelled in the particle subtracted map (yellow) with PDB **2AST** (p27 in green) and PDB **7B5R** (p27 in salmon), showing some divergence in the termini of this peptide and a potentially more helical structure of residues Asp176-Ala181.

Supplementary Table 1: Table: Statistics for Cryo-EM data collection and processing

	Hexameric Complex	SKP1-SKP2-CKS1 Particle Subtraction	CDK2-CyclinA-p27 Particle Subtraction
**Data Collection and Processing**			
Magnification		130,000 x	
Voltage (kV)		300	
Detector		Gatan K2	
Total electron exposure (e/ Å^2)		65	
Defocus range (μm)		-3.0 to -1.0 (every 0.3)	
Pixel size (Å^2)		1.07	
Total N^o movies		762	
Symmetry imposed		C1	
Initial number of particles		1,110,356	
Final number of particles		136,325	
Global resolution (Å)	3.4	3.5	3.5
FSC threshold	0.143	0.143	0.143
Map sharpening B-factor (Å^2)	121	122	126
**Refinement**			
Initial models	1JSU/2AST	2AST	1JSU
Number of atoms			
Protein	8393	4381	4834
R.M.S deviations			
Bonds (Å)	0.005	0.004	0.005
Angles (°)	0.825	0.684	0.761
Fit to map (CCmask, Phenix)	0.73	0.77	0.75
Clash score	13	9	13
Ramachandran favoured (%)	95	95	94
Ramachandran allowed (%)	5	5	6
Ramachandran outliers (%)	0	0	0
**PDB**	8BYA	8BYL	8BZO
**EMDB**	16325	16327	16344
